# Dysfunction of Wntless triggers the retrograde Golgi-to-ER transport of Wingless and induces ER stress

**DOI:** 10.1038/srep19418

**Published:** 2016-02-18

**Authors:** Peng Zhang, Lujun Zhou, Chunli Pei, Xinhua Lin, Zengqiang Yuan

**Affiliations:** 1 State Key Laboratory of Brain and Cognitive Sciences, Institute of Biophysics, Chinese Academy of Sciences, Beijing 100101, China; 2 State Key Laboratory of Biomembrane and Membrane Biotechnology, Institute of Zoology, Chinese Academy of Sciences, Beijing 100101, China; 3 Division of Developmental Biology, Cincinnati Children's Hospital Medical Center, Cincinnati, OH 45229, USA

## Abstract

Secreted Wnts play diverse roles in a non-cell-autonomous fashion. However, the cell-autonomous effect of unsecreted Wnts remains unknown. Endoplasmic reticulum (ER) stress is observed in specialized secretory cells and participates in pathophysiological processes. The correlation between Wnt secretion and ER stress remains poorly understood. Here, we demonstrated that *Drosophila miR-307a* initiates ER stress specifically in *wingless (wg)*-expressing cells through targeting *wntless (wls/evi)*. This phenotype could be mimicked by retromer loss-of-function or *porcupine (porc)* depletion, and rescued by *wg* knockdown, arguing that unsecreted Wg triggers ER stress. Consistently, we found that disrupting the secretion of human Wnt5a also induced ER stress in mammalian cells. Furthermore, we showed that a C-terminal KKVY-motif of Wg is required for its retrograde Golgi-to-ER transport, thus inducing ER stress. Next, we investigated if COPI, the regulator of retrograde transport, is responsible for unsecreted Wg to induce ER stress. To our surprise, we found that COPI acts as a novel regulator of Wg secretion. Taken together, this study reveals a previously unknown Golgi-to-ER retrograde route of Wg, and elucidates a correlation between Wnt secretion and ER stress during development.

Wnt proteins are secreted glycoproteins that regulate multiple processes during development and adult tissue homeostasis^1^. Over the last three decades, the signaling events that occur downstream of Wnt receptors have been well elucidated. However, the mechanisms underlying Wnt secretion remain largely unknown. Recent attention has been drawn to this process due to the association of aberrant Wnt levels with various diseases^2^,^3^.

Endoplasmic reticulum (ER) protein Porcupine (Porc) was the first identified regulator of Wnt secretion^4^,^5^. In *Drosophila*, Porc mediates the lipidation of Wnt proteins and facilitates their recognition by Wls^6^,^7^. Wls is a conserved transmembrane protein that regulates Wnt exocytosis from the trans-Golgi network (TGN) to the cell membrane^8^,^9^,^10^,^11^,^12^. In the absence of Wls, Wnt proteins are retained in their expressing cells. Once Wnts are exocytosed, Wls is internalized by Clathrin-mediated endocytosis^13^ and transported to TGN by the retromer complex. Retromer is a conserved multi-protein complex that sorts cargos from early endosomes (EE) back to TGN^14^. In 2008, five groups demonstrated that Wls is the target of retromer^15^,^16^,^17^,^18^,^19^. In the absence of retromer, Wls is eventually trapped into lysosomes for degradation. Later studies described an unconventional SNX3-retromer that is specifically required for Wls recycling^20^,^21^. Recently, the P24 family of proteins was identified as regulators of Wg secretion by controlling its ER export^22^,^23^. *Drosophila miR-315* was demonstrated to activate Wg signaling by targeting *axin* and *notum*^24^. *MiR-8* was reported to negatively regulate Wnt signaling at multiple levels^25^. Together, these studies indicate that the above regulators hold great potential for therapeutic targeting. However, the cell autonomous role of unsecreted Wnts is still unknown. Abnormal protein accumulation in the secretory cells leads to ER stress^26^. Upon ER stress, cells activate an integrated response, termed unfolded protein response (UPR)^27^. The ER chaperone Bip/Grp78 was upregulated upon UPR. In *Drosophila*, BiP is encoded by *hsc70-3*^28^. It is broadly expressed in embryos, with higher expression levels found in developing secretory organs^29^. Bip is a downstream target of xbp1, which is spliced and activated in response to ER stress. So, Bip is usually used as an ER marker, and its upregulation can be used an indicator of ER stress^29^. Given this information, in our genetic screen we focused on Wg secretion regulators, we also paid attention to their roles in regulating ER stress.

In this study, we identified that *miR-307a* inhibits Wg secretion through targeting *wls*. Interestingly, we found that ectopic expression of *miR-307a* initiates ER stress specifically in *wg*-expressing cells. Furthermore, we demonstrated that the C-terminal KKVY-motif of Wg mediates its Golgi-to-ER retrieval. Given that KKVY-motif is the sorting signal of the COPI complex, we investigated if COPI is responsible for unsecreted Wg to induce ER stress. Surprisingly, we found that COPI loss-of-function resulted in a Wg secretion defect and induces ER stress as well. Altogether, we discovered a novel retrograde route of Wg from the Golgi to the ER and yielded a new concept that unsecreted Wg cell-autonomously triggers ER stress.

## Results

### *MiR-307a* regulates Wg secretion and initiates ER stress in *wg*-expressing cells

**Figure 1:**
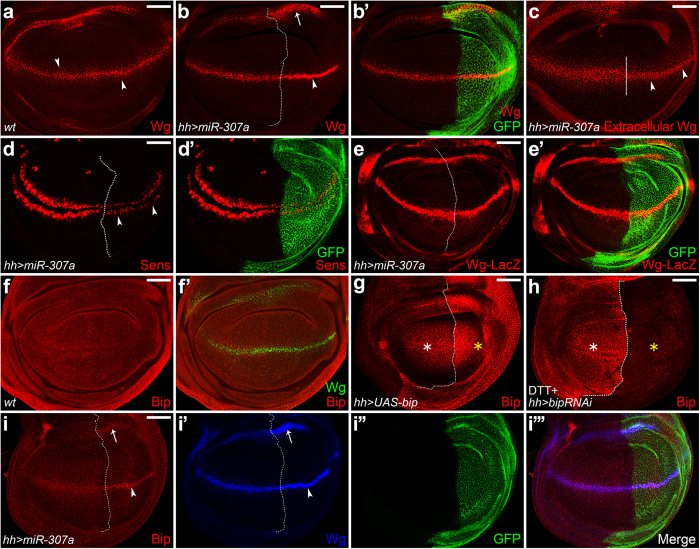
*MiR-307a* regulates Wg secretion and initiates ER stress in *wg*-expressing cells. All the wing discs hereafter are oriented anterior left, dorsal up. The dotted lines were used to indicate the Anterior/Posterior (A/P) compartment boundary. (**a**) Immunostaining of Wg in wild-type wing disc. (**b-e’, i-i”’**) Expression of *UAS-miR-307a* (marked by GFP) was induced in the P compartment using *hhGal4* driver. (**b-b’**) The arrow and arrowhead indicate Wg is accumulated in its expressing cells. (**c**) Immunostaining of extracellular Wg. The arrowheads indicate reduction of extracellular Wg staining. (**d-d’**) Immunostaining of Sens (red). The arrowheads indicate the loss of Sens expression in the *miR-307a*-expressing P compartment. (**e-e’**) Transcription of *wg* (*wg-lacZ*) is not regulated by *miR-307a* overexpression. (**f-f’**) Immunostaining of Bip and Wg in wild-type wing disc. (**g, h**) Expression of *UAS-bip* or *UAS-bipRNAi* transgene was induced using *hhGal4*. (**g**) The yellow asterisk indicates the increasing of Bip levels. (**h**) The wing discs were treated with 5 mM DTT (in M3 medium) for 16 h before immunostaining for inducing ER stress. Upregulation of Bip occurs in the wild-type tissue (white asterisk) but not in *bip* depletion tissue (yellow asterisk). (**i-i”’**) Overexpression of *miR-307a* causes an ectopic expression of Bip specifically in the *wg*-expressing cells (arrow and arrowhead in **i**). Scale bar = 50 μm.

To identify new regulators of Wg secretion, we performed a genetic screen which mainly focuses on vesicle trafficking related proteins and a microRNA library described previously^30^. The schematic representation of the *miRNA* construct was shown in (Supplementary Fig. S1a). In the screen, we identified that ectopic expression of *miR-307a* resulted in wing notches (Supplementary Fig. S1b-b’) and loss of dorsal thoracic bristles (Supplementary Fig. S1c-c’, red box in S1c’), raising the possibility that Wg signaling is disrupted. Next, we examined the role of *miR-307a* in Wg signaling in the wing imaginal discs. In the wing disc, the Wg protein is produced at the dorsal/ventral (D/V) boundary and forms a gradient along the D/V axis (Fig. 1a). Overexpression of *miR-307a* resulted in accumulation of Wg in its expressing cells (Fig. 1b-b’). The accumulation was not due to the increased transcription of the *wg* gene as the expression of *wg-lacZ* was unaffected (Fig. 1e-e’). In contrast to accumulated Wg within its expressing cells, extracellular Wg levels were reduced with *miR-307a* overexpression (Fig. 1c). Consistent with this, expression of senseless (Sens), a short-range target gene of Wg signaling^31^, is reduced by *miR-307a* overexpression (Fig. 1d-d’). Together, our data demonstrate that *miR-307a* is a negative regulator of Wg secretion.

Interestingly, we found that *miR-307a* regulates the initiation of ER stress as indicated by Bip staining. In the wild-type wing disc, Bip is ubiquitously expressed (Fig. 1f-f’). To test the specificity of the Bip antibody we used, we overexpressed *bip* by using *hhGal4* and found the staining signal of Bip antibody was obviously increased in the posterior (P) compartment (Fig. 1g). Next, we used Dithiothreitol (DTT), an ER-stress-causing agent, to treat the wing disc to elevate the basal levels of Bip (as described in^29^) since the endogenous level of Bip is relatively low (as shown in Fig.1f-f’). We found that the upregulation of Bip occurs in the wild-type anterior tissue but not in the bip-depleted posterior compartment (Fig. 1h). These experiments confirmed that the Bip antibody is sensitive enough for detecting the ectopic Bip levels. Through Bip staining, we found that overexpression of *miR-307a* by *hhGal4* caused ectopic expression of Bip specifically in the *wg-*expressing cells of the posterior wing disc (Fig. 1i-i”’).

### *MiR-307a* initiates ER stress through targeting of *wls*

**Figure 2:**
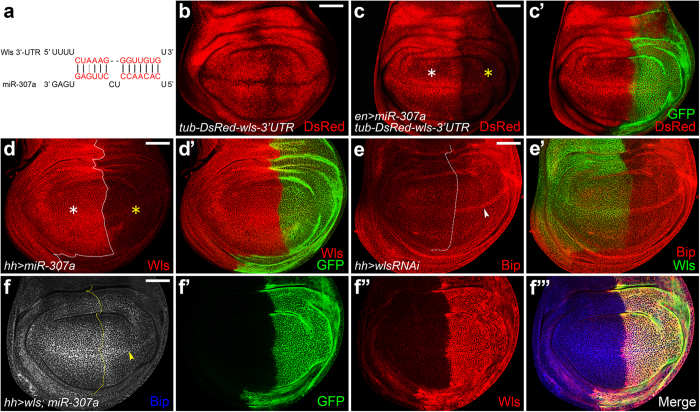
*MiR-307a* initiates ER stress through targeting Wls. (**a**) The 3’UTR of *wls* mRNA is a predicted *miR-307a* binding site. (**b**) *tub-DsRed-wls-3’UTR (wls-sensor)* was generated by cloning a 609 bp fragment of *wls 3’UTR* downstream of *pCaSpeR-tub-DsRed*. (**c-c’**) Overexpression of *miR-307a* using *enGal4* strongly inhibits the expression of *wls-sensor* (yellow asterisk in **c**). (**d-d’**) The yellow asterisk indicates the loss of *wls* expression in the *miR-307a*-expressing P compartment. (**e-e’**) *UAS-wlsRNAi* was expressed using *hhGal4*. An ectopic Bip staining was observed in the *wg*-expressing cells (arrowhead in **e**). (**f-f”’**) *UAS-wls* and *UAS-miR-307a* were co-expressed using *hhGal4*. The ectopic expression of Bip was suppressed by *wls* overexpression (arrowhead in **f**). Scale bar = 50 μm.

To investigate the possible correlation between *miR-307a* and ER stress, we searched for targets of *miR-307a* using miRanda and TargetScan^32^,^33^. We found *wls* is one of the predicted targets of *miR-307a* (Fig. 2a). To confirm this prediction, we made a *wls-sensor* transgenic fly. The expression pattern of *wls-sensor* was shown in (Fig. 2b). In the wing disc, overexpression of *miR-307a* using *enGal4* blocked expression of *wls-sensor* in the P compartment (Fig. 2c-c’). Consistently, overexpression of *miR-307a* causes a remarkable reduction in Wls protein levels (Fig. 2d-d’). These data demonstrated that *wls* is a target of *miR-307a*.

Subsequently, we generated the *miR-307a-sensor* (Supplementary Fig. S2b-b”) and *miR-307a-sponge* (Supplementary Fig. S2c) transgenes for monitoring the endogenous expression levels of *miR-307a*. Comparing with the wild-type control (Supplementary Fig. S2a), the *miR-307a-sensor* shows decreased expression in the wing pouch with striking reduction at the A/P and D/V boundaries (Supplementary Fig. S2b-b”). This pattern is similar with that of *wls-sensor*, indicating that *miR-307a* is endogenously expressed in these regions. Further, overexpression of *miR-307a-sponge* enhanced the signal of *miR-307a-sensor* (Supplementary Fig. S2d-d’). Taken together, these data suggested that *miR-307a* acts as an endogenous regulator in the wing pouch.

Next, we asked whether *miR-307a* triggered ER stress through targeting of *wls*. We found that knockdown of *wls* also induced ectopic Bip staining in the *wg*-expressing cells (Fig. 2e-e’). Moreover, overexpression of *wls* suppressed the ectopic Bip pattern induced by *miR-307a* overexpression (Fig. 2f-f”’). These data indicated that Wls acts as a negative regulator of ER stress in the *wg*-expressing cells.

### Unsecreted Wg induces ER stress in its expressing cells

**Figure 3:**
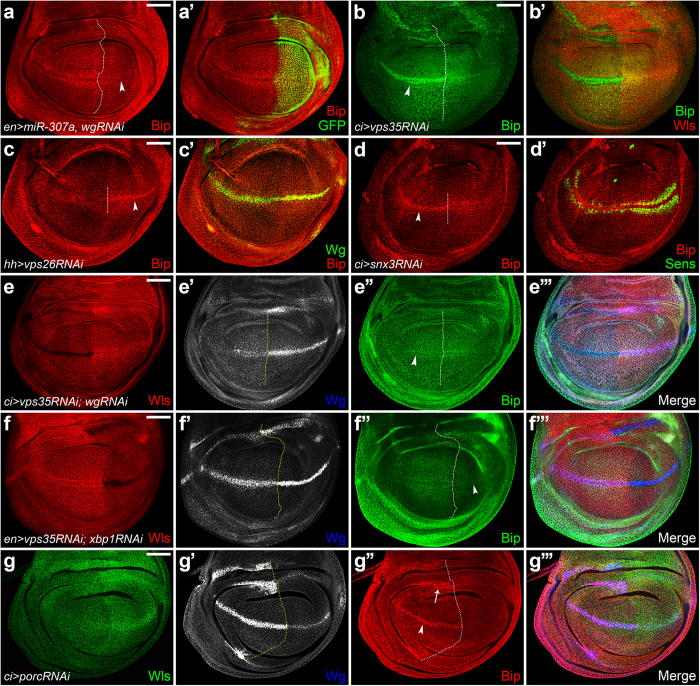
Unsecreted Wg induces ER stress in its expressing cells. (**a-a’**) *UAS-wgRNAi* and *UAS-miR-307a* were co-expressed using *enGal4. miR-307a*-induced ectopic Bip staining was suppressed by *wg* knockdown (arrowhead in **a**). (**b-b’**) *UAS-vps35RNAi* was expressed in the A compartment using *ciGal4*. Ectopic Bip staining was observed in *wg*-expressing cells (**b**, arrowhead). (**c-c’**) *vps26* RNAi was induced using *hhGal4*. Arrowhead in (**c**) indicates ectopic expression of Bip in the *wg*-expressing cells of P compartment. (**d-d’**) *snx3* RNAi was induced using ciGal4. Arrowhead in (**d**) indicates ectopic expression of Bip in the *wg*-expressing cells of A compartment. (**e-e”’**) Knockdown of *wg* in a *vps35* depletion background driven by *ciGal4*, the ectopic Bip staining was dramatically reduced (**e”**, arrowhead). (**f-f”**’) *UAS-vps35RNAi* and *UAS-xbp1RNAi* were co-expressed using *enGal4*. The ectopic Bip expression induced by *vps35* knockdown was dramatically suppressed by *xbp1* depletion (indicated by arrowhead in **f”**). (**g-g”’**) *UAS-porcRNAi* was expressed using *ciGal4*. Wg was accumulated in its expressing cells (**g’**). Bip was ectopically expressed in the *wg*-expressing cells (**g”**, arrow and arrowhead). Scale bar = 50 μm.

Given that Wls dysfunction-induced ER stress is only observed in *wg*-expressing cells, we asked whether Wg was the initiator. We found that knockdown of *wg* suppressed the ectopic expression of Bip induced by *miR-307a* (Fig. 3a-a’). *Vps35* knockdown mimicked the Bip induced phenotype caused by *miR-307a* (Fig. 3b-b’). Consistently, depleting the other components of the retromer complex, *vps26* or *snx3*, resulted in the same phenotype with *vps35* knockdown (Fig. 3c-d’). Further, knockdown of *wg* in a *vps35* depletion background efficiently inhibited the ectopic Bip expression (Fig. 3e-e”’). Moreover, increasing the ER retention of Wg by *porc* knockdown also induced ectopic Bip expression in the Wg-accumulating cells (Fig. 3g-g”’). These data demonstrated that unsecreted Wg triggers ER stress. Furthermore, depletion of *xbp1* blocked the ectopic Bip expression caused by *vps35* knockdown (Fig. 3f-f”’), suggesting that unsecreted Wg induces ER stress through the activation of Xbp1.

### Human Wnt5a acts as an ER stress initiator in mammalian cells

**Figure 4:**
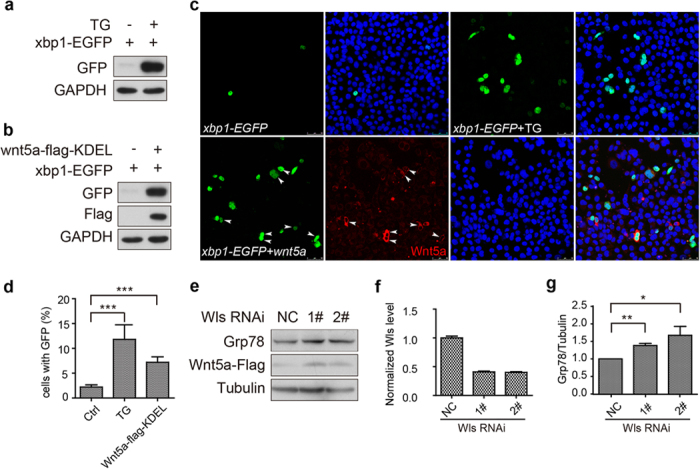
Human Wnt5a acts as an ER stress initiator in mammalian cells. (**a**) HEK293T cells were transfected with *xbp1-GFP*, and then were treated with Thapsigargin (TG, 2 μM) for 12 h at 24h post-transfection for inducing ER stress. The splicing of *xbp1* was detected by anti-GFP antibody. (**b**) HEK293T cells were co-transfected with *xbp1-GFP* and *wnt5a-flag-KDEL*. GFP and Flag antibodies were used for detecting the spliced Xbp1 and hWnt5a, respectively. (**c**) HeLa cells were transfected with *xbp1-GFP*, or together with *hWnt5a-flag-KDEL* at a ratio of 1:3. Portions of *xbp1-GFP*-transfected wells were treated with TG as positive control. The spliced Xbp1 was indicated by GFP signal. The expression of *hWnt5a* was indicated by Flag staining (red). (**d**) The cells with visible GFP expression were calculated and quantified. Data represent the Mean ± SEM (*t*-test, ***P < 0.001, n = 10 random selected fields). (**e**) HEK293T cells that stably expressed *hWnt5a-Flag* were transfected with *hWls* siRNA. The transfected cells were washed twice at 72hr post-transfection for lysis. Lysate was immunobloted with Grp78, Flag and Tubulin antibodies. (**f**) The knockdown efficiency of *hWls* siRNAs was detected by real-time PCR. (**g**) The relative levels of GRP78 as shown in (**e**) were quantified after normalization against Tubulin. Values represent the Mean ± S.E.M. (student’s *t*-test, n = 3, *P < 0.05, **P < 0.01).

Next, we investigated whether the Wg-induced ER stress is conserved in mammalian cells. We generated a *xbp1-GFP* reporter for ER stress as described in^34^. In metazoans, a 26-nucleotide intron of *xbp1* mRNA is spliced out during ER stress, causing a shift in the codon reading frame^34^. The spliced *xbp1* mRNA is translated into the mature XBP1 protein in response to ER stress^34^. In this case, we fused the gene encoding GFP downstream of a partial sequence of human *xbp1*, including the 26-nt ER stress-specific intron. Under normal conditions, the mRNA of the fusion gene would not be spliced, and that its translation would be terminated at the stop codon near the joint between the XBP1 and GFP. During ER stress, the 26-nt intron should be spliced out. Thus, a fusion protein of XBP1-GFP should be produced in cells. To test whether *xbp1-GFP* works as an indicator for ER stress, we transfected HEK293T cells with *xbp1-GFP*, and then treated them with Thapsigargin (an agent that promotes ER stress by depletion of lumenal calcium storage). The splicing of *xbp1* was detected by anti-GFP antibody (Fig. 4a) or GFP fluorescence (the upper panels of Fig. 4c), indicating that the *xbp1-GFP* works well. Next, We co-transfected HeLa cells with *xbp1-GFP* and *hwnt5a-flag-KDEL* (an ER retention form of Wnt5a). A remarkable splicing of *xbp1* was observed (Fig. 4b and the lower panels of Fig. 4c). Through quantification, we found the percentage of cells with visible GFP expression was significantly increased by *hwnt5a-flag-KDEL* overexpression (Fig. 4d). These data indicated that ER stress is triggered. Furthermore, Knockdown of *wls* in HEK293T cells which stably expressed *hWnt5a*. The knockdown efficiency of two Wls RNAi could reach 50% (Fig. 4f). The lysates were immunobloted with Flag antibody. The amount of Wnt5a in cell lysates was increased upon Wls knockdown (Fig. 4e, middle panel), indicating that Wnt5a was accumulated in the cells. Also, an upregulation of Grp78 was observed (up panel of Fig. 4e,g). Taken together, these data suggested that hWnt5a act as an initiator of ER stress when its secretion route is disrupted.

### A C-terminal KKVY-motif mediates the Golgi-to-ER retrieval of Wg

**Figure 5:**
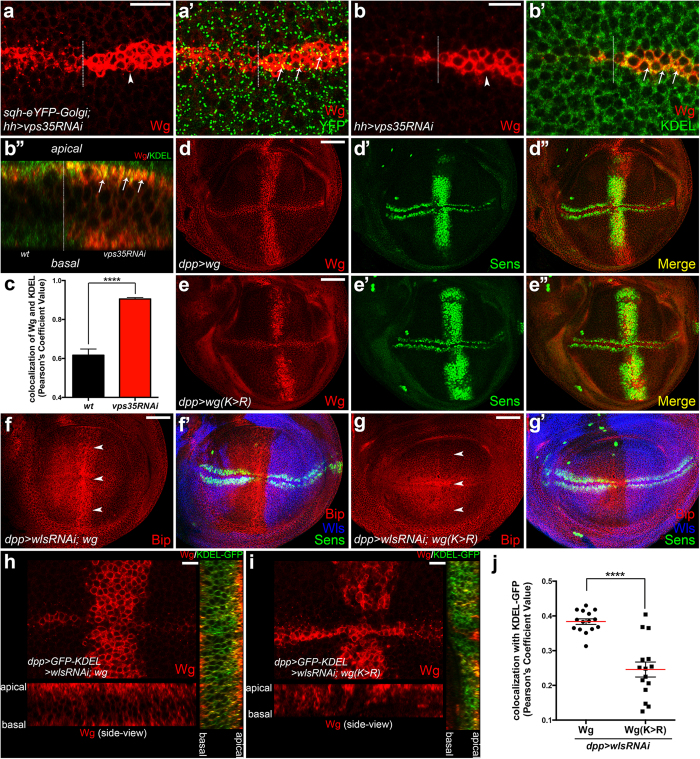
A C-terminal KKVY-motif of Wg mediates its Golgi-to-ER retrieval. (**a-a’**) *UAS-vps35RNAi* was driven by *hhGal4* carrying *sqh-eYFP-Golgi* transgene. Wg was accumulated in the posterior *wg*-expressing cells (arrowhead in **a**). The colocalization of unsecreted Wg with Golgi marker was mildly enhanced (arrows in **a’**) compared with the wild-type control. (**b-b”’**) *UAS-vps35RNAi* was expressed using *hhGal4*. The unsecreted Wg shows increasing of colocalization with the ER marker, KDEL (arrows in **b’**,**b”**). (**b”**) shows the side-view of Wg-expressing cells. Z-section images were taken from apical to basal of (**b**). (**c)** Cross sections were cut along the Wg-expressing region for colocalization analysis. The Pearson’s Correlation (PC) value represents the colocalization of Wg with KDEL. The colcalization of unsecreted Wg with KDEL was significantly increased compared with the wild-type control. Data represent the Mean ± SEM (*t*-test, n = 10, ****P < 0.0001). (**d-e”**) Ectopic expression of Sens can be induced by either *UAS-wg* or *UAS-wg(K334K > R334R)* driven by *dppGal4*. (**f-g’**) *UAS-wg* or *UAS-wg(K334K > R334R)* was co-expressed with *UAS-wlsRNAi* using *dppGal4*, respectively. The genetic crosses and immunostaining were performed under the same condition. Images were taken with same laser setting on the confocal microscope. The accumulation of wild-type Wg in its expressing cells induces remarkable ectopic Bip expression (arrowheads in **f**). Whereas, the K-to-R mutant form of Wg does not show obvious induction of ectopic Bip (arrowheads in **g**). (**h-i**) *UAS-wg* or *UAS-wg(K334K > R334R)* was co-expressed with *UAS-wlsRNAi* and *UAS-GFP-KDEL* using *dppGal4*, respectively. Z-stack sections were taken with same laser setting on the confocal microscope. The subcellular distribution of Wg or Wg(K > R) was shown in the lower panel. The colocalization of Wg or Wg(K > R) with GFP-KDEL was shown in the right panel. (**j**) Cross sections were cut along the *dppGal4*-expressing region for colocalization analysis. Comparing with the wild-type Wg, the K-to-R mutant form of Wg shows weaker colocalization with GFP-KDEL. Data represent the Mean ± SEM (*t*-test, n = 15, ****P < 0.0001). The Wg dilution used in this Figure was 1:20. Scale bars in (**a,b,h,i**) 10 μm; (**d–g**) 50 μm.

As Wls exports Wg from Golgi to the cell membrane, we asked why Wls dysfunction could induce ER stress. In the *vps35-depleted wg*-expressing cells, we observed the subcellular co-localization of Wg with a Golgi marker was enhanced (Fig. 5a-a’). Interestingly, an increased co-localization of Wg with an ER marker was also observed (Fig. 5b,c and Supplementary Fig S3a,b). We hypothesized that the transient accumulation of Wg in Golgi triggers its retrieval to the ER. Previous studies demonstrated that COPI complex retrieves the cargos containing the canonical C-terminal KKxx- or KxKxx-motif as well as the non-canonical KxHxx- or RKxx-motif^35^,^36^. Coincidently, there is a KKVY-motif in the C-terminal of Wg. We generated the *UAS-wg(K*^*334*^*K > R*^*334*^*R)* and *UAS-wg* transgenic flies using the PhiC31 integrase-mediated site-specific transgenesis system. Both of these are integrated into the *3R 86F* locus to ensure that at least the transcripts have the same expression levels. We found that the K-to-R mutant form of Wg maintained its signaling transduction ability (Fig. 5d,e), indicating its secretion and transporting functions were unaffected under normal conditions. Next, we co-expressed *UAS-wg* and *UAS-wg(K*^*334*^*K > R*^*334*^*R)* with *UAS-wlsRNAi* using *dppGal4*. In this context, we found the unsecreted wild-type Wg caused obvious ER stress (Fig. 5f-f’). However, the phenotype was not observed in the mutant form of Wg (Fig. 5g-g’). Furthermore, the K-to-R mutant form of Wg showed weaker colocalization with the ER marker (Fig. 5h–j) but stronger colocalization with the Golgi marker (Supplementary Fig. S3c–e) compared with the wild-type of Wg. Taken together, these data suggested that the KKVY-motif mediates the retrieval of unsecreted Wg from Golgi to ER, thus inducing ER stress.

### COPI regulates Wg secretion and ER stress initiation

**Figure 6:**
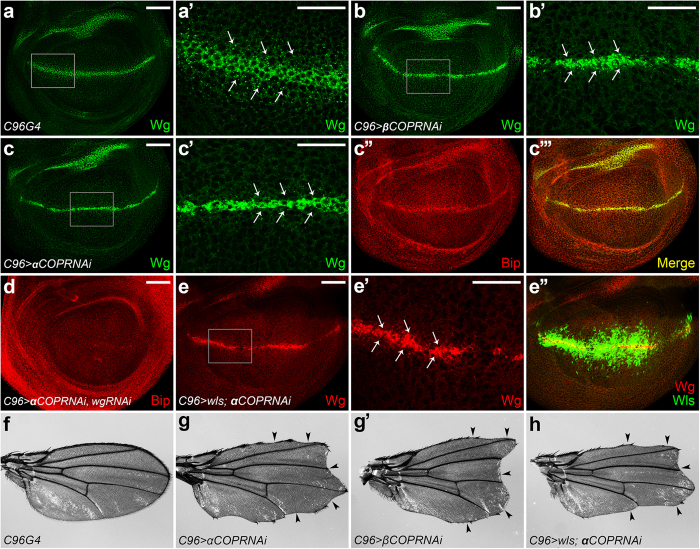
COPI regulates Wg secretion and ER stress initiation. (**a**) Wg staining in the wild-type wing disc. (**a’**) Enlarged view of the gray box region in (**a**). (**b-b’**) *UAS-βCOPRNAi* was expressed using *C96Gal4*. Wg secretion was disrupted by *βCOP* knockdown. The secreted punctate structures of Wg were dramatically reduced (indicated by arrows in **b’**). (**c-c”’**) Depletion of *αCOP* using *C96Gal4* generated same phenotype (shown in **c’**). Ectopic Bip induction was also observed along the D/V boundary (shown in **c”**). (**d**) The ectopic Bip expression was suppressed by *wg* knockdown. (**e-e”**) *UAS-wls* and *UAS-αCOPRNAi* were co-expressed by *C96Gal4* driver. *αCOP* depletion-induced Wg secretion defect cannot be rescued by *wls* overexpression (arrows in **d’**). (**f)** Adult wing of *C96Gal4*. (**g-g’**) *UAS-αCOPRNAi* or *UAS-βCOPRNAi* was expressed using *C96Gal4*. Knockdown either of them induced obvious wing notches (indicated by arrowheads in **d’,d”**). (**h**) Overexpression of *wls* failed to rescue the wing notching phenotype induced by *αCOP* knockdown. Scale bars in (**a–e)** 50 μm; (**a’,b’,c’,e**’) 20 μm.

Since COPI is the key regulator of the retrograde Golgi-to-ER transport, we asked if COPI is responsible for unsecreted Wg to induce ER stress. To investigate this, we performed the epistasis tests between Wls and the COPI subunits, αCOP and βCOP^37^, respectively. Surprisingly, knockdown of either *αCOP* or *βCOP* alone could induce Wg secretion defect (Fig. 6a-c”’). Consistently, the adult flies displayed notched wings (Fig. 6f-g’), suggesting the deficient of Wg signaling. In addition, an ectopic Bip expression was observed in the *αCOP*-depleted *wg*-expressing cells (Fig. 6c”). This phenotype could be suppressed by *wg* knockdown (Fig. 6d), suggesting that COPI regulates ER stress initiation through controlling Wg secretion. Taken together, these data revealed a novel role for COPI that regulated Wg secretion and ER stress initiation as well.

## Discussion

Wnt secretion and ER stress are two fundamental biological processes that participate in diverse biological and pathophysiological processes. The correlation between these processes remains poorly understood. Wnt proteins play roles in a cell non-autonomous fashion. However, the cell-autonomous effect of unsecreted Wnts is still unknown. It has been reported that deletion of *p24* genes activates an ER stress response which alleviates the deleterious effects of the *p24* deletion^38^,^39^. As the P24 family of proteins controls Wg secretion by regulating the ER export of Wg^22^,^23^, this data led us to pursue the possibility that unsecreted Wg triggers ER stress. Our new findings yielded a new concept that unsecreted Wg can cell-autonomously trigger ER stress.

In this study, we identified that *miR-307a* inhibits Wg secretion through targeting *wls*. Intriguingly, we found that overexpression of *miR-307a* induces ER stress specifically in the *wg*-expressing cells. This phenotype could be mimicked by retromer loss-of-function or *porc* depletion and could be rescued by *wg* knockdown, suggesting that unsecreted Wg triggers ER stress. We hypothesized that the transiently Golgi-accumulated Wg was retrieved to ER thereby inducing ER stress. Previous studies have demonstrated that COPI governs the retrograde Golgi-to-ER transport by recognition of the C-terminal KKxx-motif of type I transmembrane proteins^35^,^36^. Coincidently, there is a KKVY sequence in the C-terminal of Wg. By further *in vivo* assay, we found that the K-to-R mutant form of Wg lost the ability to trigger ER stress, suggesting that the KKVY-motif of Wg is required for its retrieval and ER stress induction. Since Wg is a protein on the luminal side, it is most unlikely that Wg was directly recognized by COPI. Given this, we suppose that there could be another transmembrane bridge protein(s) mediating the interaction of Wg with COPI. Through the alignment of *Drosophila* P24 family of proteins, we found a typical COPI-recognized KKxx-motif (KKLV) in the C-terminal of Éclair (Eca). Interestingly, it has been demonstrated that Eca is required for the ER-to-Golgi anterograde of Wg^23^. Together, it raised a possibility that Eca is retrieved back to the ER by COPI for reuse. The interaction between Eca and Wg may facilitate the retrieval of Wg. However, whether the KKVY-motif of Wg mediates its interaction with Eca in the retrieval route needs to be further investigated.

Next, we tested whether knockdown of COPI can suppress the ER stress caused by *wls* depletion. But, we surprisingly found that downregulation of COPI unexpectedly disrupted Wg secretion and triggered ER stress. This phenotype raised two possibilities: First, the regulators that control the ER-to-Golgi transport of Wg might be retrieved to the ER for reuse by COPI (as discussed above). In this case, COPI loss-of-function could block the retrieval of these regulators, thereby indirectly inhibiting Wg secretion and inducing ER stress. Second, Wls is retrieved from Golgi to ER by COPI to facilitate Wg secretion. In this case, COPI loss-of-function will block the retrieval of Wls, thus increasing the ER retention of newly synthesized Wg and initiating ER stress. The second possibility was supported by a recent study from Virshup lab. In that study, they identified a C-terminal RKEAQE-motif of human Wls that can be recognized by COPI for retrieval^40^. However, this motif is not very conserved in *Drosophila* Wls. Interestingly, they showed that Wls overexpression rescued the effects of *ERGIC2* (a subunit of vertebrate COPI) depletion in *X. laevis* embryos^40^. However, in *Drosophila*, we found that Wls overexpression failed to rescue the Wg secretion defect induced by *αCOP* RNAi (Fig. 6e-e”), which might be due to general effect upon COPI depletion. Alternatively, Wls might require COPI for the function. In addition, previous study has shown that Wg colocalized with Golgi-localized Wls^18^, and the Golgi-localization of the EE-to-TGN recycled Wls depends on the levels of Wg^18^. These data suggested that Wls encounters with Wg predominantly in the Golgi apparatus and the stability of Wls might be depended on its interaction with Wg. Moreover, if Wls is indeed retrieved to the ER to bind Wg, blocking the ER export of Wg should not influence the interaction of Wls with Wg. However, blocking the ER export of Wg by knockdown of P24 proteins reduced the punctuate pattern of Wls in *wg*-expressing cells^23^, suggesting that the interaction of Wls with Wg was decreased. Consistently, we found that increasing the ER retention of Wg by *porc* knockdown also reduced the Wls levels (Fig. 3g). These data suggested consistent with the idea that *Drosophila* Wls may not be retrieved to the ER by COPI. The molecular mechanism regarding the *Drosophila* Wls retrieval *in vivo* needs to be further investigated.

Several components of the UPR have been shown to play a protective role against the progression of disease^41^. In this study, we found that Wls dysfunction-induced ER stress buffers the toxicity of unsecreted Wg during development, while the detailed mechanisms need to be further addressed. Additionally, previous study reported that tumour hypoxia blocks Wnt secretion through inducing ER stress^42^. Together with our findings, whether a feedback loop exists between Wnt secretion and ER stress remains to be elucidated. Mutations in human *porcupine (porcn)* cause the X-linked dominant disorder focal dermal hypoplasia (FDH, also known as Goltz syndrome)^43^,^44^. Vps35, a component of the retromer complex, has been found to be often mutated in PD patients^45^,^46^. Whether unsecreted Wg-induced ER stress plays a role in these diseases is a promising topic. In summary, we discovered a retrograde route of Wg from the Golgi to the ER and elucidated a correlation between Wnt secretion and ER stress. Given the critical roles of aberrant Wnt signaling and ER stress in the pathogenesis of human diseases, identifying regulators that connect these two processes will facilitate approaches for therapeutic intervention.

## Methods

### *Drosophila* strains

The *enGal4, hhGal4, apGal4, ciGal4, dppGal4, C96Gal4* and *wg-lacZ* were as described in FlyBase. The *UAS-wlsRNAi* (103812), *UAS-wlsRNAi* (5214), *UAS-porcRNAi* (47864), *UAS-snx3RNAi* (104494), *UAS-vps26RNAi* (18396), *UAS-αCOPRNAi* (35305) and *UAS-βCOPRNAi* (15418) were obtained from the Vienna *Drosophila* RNAi Center. The *UAS-bipRNAi* (HMS00397), *UAS-xbp1RNAi* (HMS03015) and *UAS-wgRNAi* (HMS00844) were obtained from the *Drosophila* RNAi Screen Center at Harvard Medical School. *UAS-bip* (5843), *sqh-eYFP-golgi* (7193), *sqh-eYFP-ER* (7195), *UAS-GFP-KDEL* (9898) and *UAS-GRASP65-GFP* (8507) was obtained from Bloomington *Drosophila* Stock Center. *UAS-vps35RNAi* and *UAS-wls* were described previously^15^. *UAS-GFP-miR-307a, UAS-DsRed-miR-307a-sponge, UAS-wg, UAS-wg(K*^*334*^*K > R*^*334*^*R), tub-EGFP, tub-EGFP-miR-307a-sensor* and *tub-DsRed-wls-3*′*UTR* transgenes were generated in this study.

### Plasmid construction

To generate the *pWALIUM10-moe-GFP-GPI-miR-307a*, 714 bp of genomic DNA surrounding *miR-307a* was amplified by PCR and cloned downstream of GFP-GPI in the NheI site of *pWALIUM10-moe-GFP-GPI vector*^30^.

Forward: 5′-CGGCTAGCCGGAACGAGGATTCTG-3′

Reverse: 5′-CGGCTAGCCCTGATGGTTTAAGTCCTG-3′

To generate the *pWALIUM10-moe-DsRed-miR-307a-sponge*, the following sequence was designed as described in^47^, and then directly cloned into *pWALIUM10-moe-DsRed* through BglII and NdeI sites. The sequence is:

5′-AGATCTCTCACTCAGATGGTTGTGAAATCCTCACTCAGATGGTTGTGAAATCCTCACTCAGATGGTTGTGAAATCCTCACTCAGATGGTTGTGAAATCCTCACTCAGATGGTTGTGAAATCCTCACTCAGATGGTTGTGAAATCCTCACTCAGATGGTTGTGAAATCCTCACTCAGATGGTTGTGACATATG-3′

To generate the *pCaSpeR-tub-EGFP-miR307a-sensor*, the following primers were annealed and then cloned into *pCaSpeR-tub-EGFP* vector (a gift from T. Kai) through NotI and XhoI sites. The primers are:

Forward: 5′-GGCCGCCTCACTCAAGGAGGTTGTGAAATCACACCTCACTCAAGGAGGTTGTGAC-3′

Reverse: 5′-TCGAGTCACAACCTCCTTGAGTGAGGTGTGATTTCACAACCTCCTTGAGTGAGGC-3′

To generate the *pCaSpeR-tub-DsRed-wls-3*′*UTR*, a 609 bp fragment of the Wls 3′UTR was amplified by PCR from wild-type genomic DNA and cloned downstream of *pCaSpeR-tub-DsRed* through NotI and XhoI sites. The primers are:

Forward: 5′-AAAGCGGCCGCGCGGAAGGACTCGAATTATTG-3′

Reverse: 5′-GGGCTCGAGCAATATTGCTTTTTATTCGATGCA-3′

To generate the *UAS-wg* construct, the full-length cDNA of *wg* was cloned into the *pUAST-attB* vector through NotI and XhaI sites. The primers are:

Forward: 5′-AAAGCGGCCGC ATGGATATCAGCTATATCTTCGTC-3′

Reverse: 5′-GGGTCTAGATTACAGACACGTGTAGATGACC-3′

A similar strategy was used to make the *UAS-wg(K*^*334*^*K > R*^*334*^*R)*. The point mutation was generated by PCR using the following primers:

Forward: 5′-GCTGTGTCGGACCCGACGGGTCATCTACACGTGTCTGTAATCTAG-3′

Reverse: 5′-CACGTGTAGATGACCCGTCGGGTCCGACACAGCTTGCACTTCACCTCG-3′

To generate the *pEGFP-N1-xbp1*, a 596 bp fragment of xbp1 (from 49 to 644) was cloned into *pEGFP-N1* through EcoRI and BamHI sites. The primers are:

Forward: 5′-GGGGAATTCATGGTGGTGGTGGCAGCC-3′

Reverse: 5′-AAAGGATCCCGTGAATCTGAAGAGTCAATACCGCC-3′

To generate *pSIN-wnt5a-flag*, primers containing flag sequence were designed to amplify *wnt5a* from the plasmid *pcDNA3.1-wnt5a* (a gift from Q. Tao). The primers are:

Forward: 5′-AAAGAATTCTTACAGTTCATCCTTCTTGCACACAAACTGGTCCAC-3′

Reverse: 5′-AAAGAATTCTTACAGTTCATCCTTCTTGCAGGTGTGCACGTCG-3′

Similar strategy was used to generate the *pcDNA3.1-wnt5a-flag-KDEL*. The primers used are:

Forward: 5′-GGGACTAGTATGGCTGGAAGTGCAATGTC-3′

Reverse: 5′-AAAGGATCCTCACTTATCGTCGTCATCCTTGTAATCCTTGCACACAAACTGGTCCAC-3′

### Cell culture, transfection and western blot

HEK293T and HeLa cell lines were cultured in DMEM supplemented with 10% fetal bovine serum (Gibco), 50U/ml penicillin, 50 μg/ml streptomycin, in 5% CO_2_ atmosphere at 37 °C. Plasmid transfection was carried out using LipofectAMINE (Invitrogen) according to manufacturer’s instructions. 2 μM Thapsigargin (Enzo Life Sciences) was used to induce ER stress. The primary antibodies used for western blot were rabbit anti-Grp78 (Cell Signaling Technology), mouse anti-Flag (Sigma), rabbit anti-GFP (Invitrogen) and mouse anti-β-Tubulin (CWBiotech).

### Immunostaining and microsopy

Antibody staining of wing imaginal discs or cells was performed using standard protocols. The following primary antibodies were used: rat anti-Bip (MAC143, Abcam), mouse anti-Wg (4D4; DSHB), guinea pig anti-Sens^31^, rabbit anti-Wls^21^, rabbit anti-GFP Alexa Fluor 488 (Molecular Probe), mouse anti-lacZ (Abmart), Rat anti-KDEL (Abcam), and Rabbit anti-GM130 (Abcam). The nuclei were stained by Hoechst 33258 (Sigma). The primary antibodies were detected by fluorescent-conjugated secondary antibodies from Jackson ImmunoResearch Laboratories. Confocal images were collected using a Lecia TCS SP5 confocal microscope with 40X/1.25 oil objectives. Adult thoracic bristle and wing images were obtained using a Nikon SMZ1500 microscope. Z-section images were taken with a Zeiss LSM 780 confocal microscope with 40X/1.3 oil objectives for the colocalization analysis. For determination of the Pearson’s correlation coefficient, the JACoP plugin^48^ for ImageJ was applied. Images were processed with ImageJ and Adobe Photoshop.

### Statistical analysis

Statistical analyses were performed using the Graphpad Prism 6 software package. Statistical significance (P values) of the results was calculated by unpaired two-tailed Student’s t test. Data are presented as the Mean ± SEM, *P < 0.05, **P < 0.01, ***P < 0.001 and ****P < 0.0001 denote statistical significance.

## Additional Information

**How to cite this article**: Zhang, P. *et al.* Dysfunction of Wntless triggers the retrograde Golgi-to-ER transport of Wingless and induces ER stress. *Sci. Rep.* **6**, 19418; doi: 10.1038/srep19418 (2016).

## Supplementary Material

Supplementary Information
