# Supplementary material for: Dysfunction of Wntless triggers the retrograde Golgi-to-ER transport of Wingless and induces ER stress
Source: Sci Rep. 2016 Feb 18;6:19418. doi: 10.1038/srep19418 (PMC4757895; doi:10.1038/srep19418)
Supplement: Supplementary Information [file srep19418-s1.pdf]

Supplementary file

Dysfunction of Wntless triggers the retrograde Golgi-to-ER transport of Wingless and induces ER stress

Peng Zhang [1], Lujun Zhou[1], Chunli Pei[1], Xinhua Lin[2] & Zengqiang Yuan[1,※]

[1]State Key Laboratory of Brain and Cognitive Sciences, Institute of Biophysics, Chinese Academy of Sciences, Beijing 100101, China

[2]State Key Laboratory of Biomembrane and Membrane Biotechnology, Institute of Zoology, Chinese Academy of Sciences, Beijing 100101, China; Division of Developmental Biology, Cincinnati Children's Hospital Medical Center, Cincinnati, OH 45229, USA

※Correspondence should be addressed to Z.Y. (zqyuan@ibp.ac.cn)

Supplementary figures

Supplementary figure S1. Expression of *miR-307a* blocks Wg signaling.

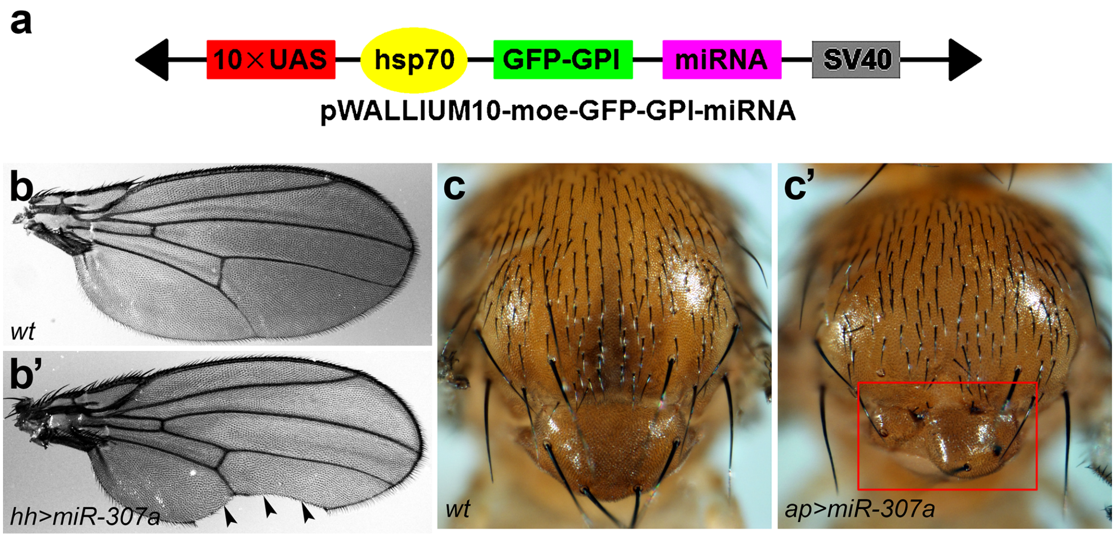

(a) Schematic representation of the pWALIUM10-moe-GFP-GPI-miRNA construct.

(b) Adult wing of *hhGal4*. (b') Overexpression of *miR-307a* using *hhGal4* driver causes obvious notching at the posterior margin region. (c-c') Expression of *miR-307a* in the dorsal compartment using *apGal4* results in obvious loss of dorsal thoracic bristles (c', red box) compared with the *wild-type* control (c).

Supplementary figure S2. *miR-307a* acts an endogenous regulator in the wing disc.

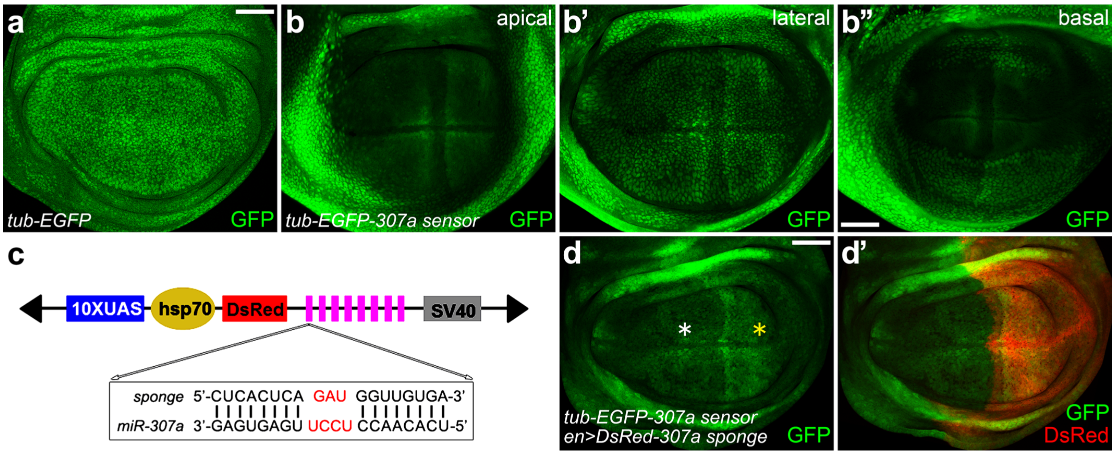

(a) Expression pattern of *tub-EGFP* in wing disc. (b-b'') Two complementary sites with perfect matches for *miR-307a* were placed downstream of *pCaSpeR-tub-EGFP* as *miR-307a-sensor*. The expression of *miR-307a-sensor* was uniformly reduced in the wing pouch, especially at the D/V and A/P boundary region. (b), (b') and (b'') showed the apical, lateral and basal sections of the wing disc, respectively. (c) Schematic representation of the *miR-307a-sponge (miR-307a-sp)*. Eight repeats of the designed sequence were tandemly placed downstream of *pWALIUM10-moe-DsRed*. (d-d') Overexpression of *miR-307a-sponge* using *enGal4* leads to increased levels of miR-307a-sensor in the posterior compartment (yellow asterisk in d). The cross was set up at 29℃. Scale bar=50 μ m.

Supplementary figure S3. Unsecreted Wg was retrograded to the ER dependent of its C-terminal KKVY-motif.

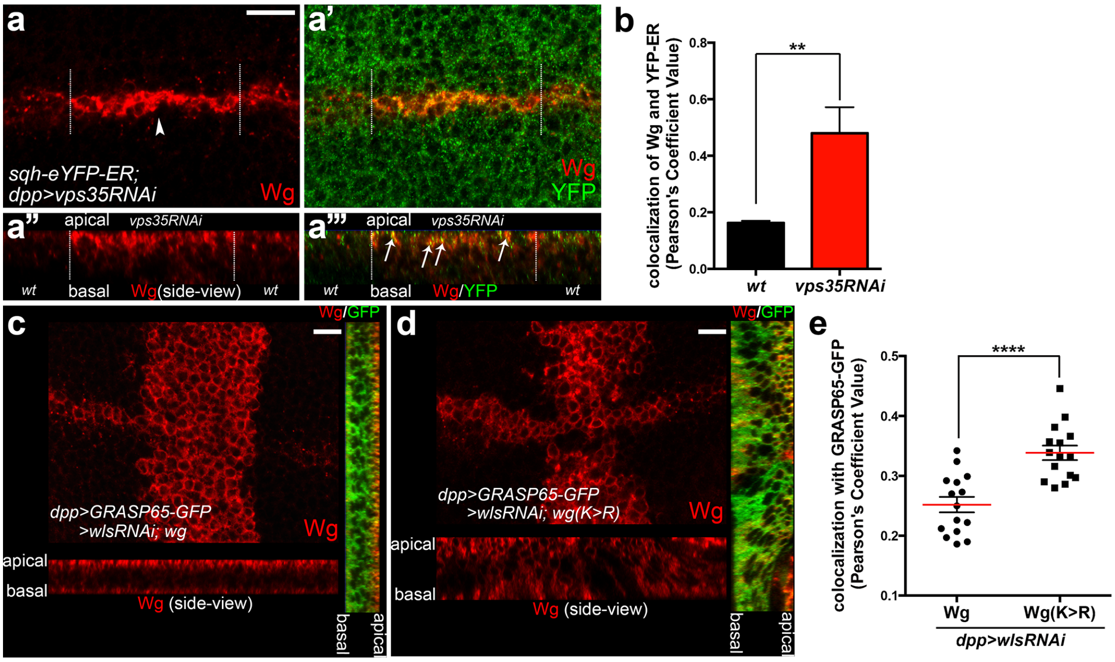

(a-a') *UAS-vps35RNAi* was driven by *dppGal4* carrying *sqh-eYFP-ER* transgene. Wg was significantly accumulated in the *vps35*-depleted cells (arrowhead in a). The unsecreted Wg shows increased colocalization with ER marker (green) compared with the wild-type control. Z-stack sections were taken from apical to basal of (a'). (a''-a''') shows the cross section of the Wg-expressing cells. The dotted line labels the boundary of *vps35*-depleting region. (a''') Arrows indicate the colocalization of Wg with YFP-ER. (b) Cross sections were cut along Wg-expressing region for the colocalization analysis. The Pearson's Correlation Value represents the colocalization of Wg with YFP-ER. The *vps35*-depleted cells show increased colcalization of Wg with YFP-ER. Data represent the Mean ± SEM (*t*-test, n=5, ** $P < 0.01$). (c-d) *UAS-wg* or *UAS-wg(K334K>R334R)* was co-expressed with *UAS-wlsRNAi* and *UAS-GRASP65-GFP* using *dppGal4*, respectively. Z-stack sections were taken with

same laser setting on the confocal microscope. The subcellular localization of Wg or Wg(K>R) was shown in the lower panel. The colocalization of Wg or Wg(K>R) with GRASP654-GFP was shown in the right panel. (e) Cross sections were cut along the *dppGal4*-expressing region for colocalization analysis. Comparing with the wild-type of Wg, the K-to-R mutant form of Wg shows stronger colocalization with GRASP65-GFP. Data represent the Mean ±SEM (*t*-test, n=15, **** $P < 0.0001$). The Wg dilution used in this Figure was 1:20. Scale bar=10 μ m.
